# No Modulatory Effects when Stimulating the Right Inferior Frontal Gyrus with Continuous 6 Hz tACS and tRNS on Response Inhibition: A Behavioral Study

**DOI:** 10.1155/2018/3156796

**Published:** 2018-10-23

**Authors:** Hannah Brauer, Navah Ester Kadish, Anya Pedersen, Michael Siniatchkin, Vera Moliadze

**Affiliations:** ^1^Institute of Medical Psychology and Medical Sociology, University Hospital of Schleswig-Holstein (UKSH), Campus Kiel, Christian Albrechts University, Kiel, Germany; ^2^Department of Child and Adolescent Psychiatry and Psychotherapy, School of Medicine, Christian Albrechts University, Kiel, Germany; ^3^Clinical Psychology and Psychotherapy, Christian Albrechts University, Kiel, Germany

## Abstract

Response inhibition is the cognitive process required to cancel an intended action. During that process, a “go” reaction is intercepted particularly by the right inferior frontal gyrus (rIFG) and presupplementary motor area (pre-SMA). After the commission of inhibition errors, theta activity (4–8 Hz) is related to the adaption processes. In this study, we intend to examine whether the boosting of theta activity by electrical stimulation over rIFG reduces the number of errors and the reaction times in a response inhibition task (Go/NoGo paradigm) during and after stimulation. 23 healthy right-handed adults participated in the study. In three separate sessions, theta tACS at 6 Hz, transcranial random noise (tRNS) as a second stimulation condition, and sham stimulation were applied for 20 minutes. Based on behavioral data, this study could not show any effects of 6 Hz tACS as well as full spectrum tRNS on response inhibition in any of the conditions. Since many findings support the relevance of the rIFG for response inhibition, this could mean that 6 Hz activity is not important for response inhibition in that structure. Reasons for our null findings could also lie in the stimulation parameters, such as the electrode montage or the stimulation frequency, which are discussed in this article in more detail. Sharing negative findings will have (1) positive impact on future research questions and study design and will improve (2) knowledge acquisition of noninvasive transcranial brain stimulation techniques.

## 1. Introduction

Response inhibition, as an important process of executive control, refers to the suppression of actions that are no longer required or that are inappropriate. It allows flexible and goal-directed behavior in ever-changing environments. The computational routine during response inhibition is well described [1, 2]. It is related to a dynamic information flow between the right inferior frontal cortex (rIFC) and the presupplementary motor area (pre-SMA) through basal ganglia to the primary motor cortex (M1) (for review, see [2–4]). In this network, pre-SMA and IFC seem to play a driving function regulating performance monitoring, continuous preparation of actions, and attentional control. Especially the rIFG (right inferior frontal gyrus) is an important structure for motor response inhibition. Lesion studies with transcranial magnetic stimulation (TMS) show that deactivation of the pars opercularis of the rIFG leads to more inhibition errors in a Go/NoGo task (e.g., [5]). Using the Go/NoGo task, Mazaheri and colleagues [6] demonstrated that errors during response inhibition can be predicted by theta-alpha coupling in healthy adults: after an error, there is a significant increase in theta power in pre-SMA and IFC and decrease of alpha power in the parieto-occipital cortex (POC) following an error and preceding a successful response. In children with ADHD, lower theta-alpha coupling was seen in comparison to healthy children [7]. It may be hypothesized that the increase in theta activity in pre-SMA and IFC would reduce the number of errors and improve inhibitory control.

Noninvasive transcranial brain stimulation (NTBS) techniques such as TMS and transcranial electrical stimulation (tES) are important tools in human systems and cognitive neuroscience which are able to modulate activity in the neural tissue underlying the stimulating area. To date, the majority of studies in humans use transcranial direct current stimulation (tDCS) to modulate cortical function. It is thought that tDCS is capable of inducing polarity-dependent, relatively long-lasting changes in the human brain (for recent reviews, see [8–11]). The most relevant for our study is that Jacobson and colleagues [12] and Cunillera and colleagues [13] described that anodal tDCS to the rIFG improves behavioral inhibition, suggesting that tDCS modulates cognitive control in healthy individuals. However, tDCS does not allow to investigate whether this modulation is related to specific frequencies.

Numerous studies have demonstrated that transcranial alternating current stimulation (tACS) provides the unique possibility of noninvasively modulating ongoing oscillatory activity in a frequency-specific way, which has been attributed to oscillatory entrainment by the specific stimulation frequency [14–17] (for review, see Antal and Herrmann [18]). Besides changing the frequency of oscillations, tACS is also able to enhance the power of a certain frequency band. Zaehle and colleagues [14] elevated endogenous alpha power in parieto-central regions with individual alpha frequency stimulation.

Recent studies on cognitive processes in both young and older healthy subjects indicate successful modulation of brain oscillations and behavioral outcome through frontal or parietal tAC stimulation. The majority of the studies suggest a particularly beneficial effect of tACS in the theta frequency [19–21]. Polania and colleagues [16] demonstrated that the tACS with 6 Hz frequency over the prefrontal cortex (DLPFC) can boost not only activity in the DLPFC but also in the parietal cortex, increasing working memory capacity. The authors provide evidence that tACS in the theta frequency range applied to the prefrontal cortex is able to increase frontoparietal connectivity and improve neuropsychological function. Further study confirmed the beneficial effect of theta tACS on accuracy in verbal working memory [22]. Pahor and Jausovec [23] observed reduced alpha power in posterior areas after theta tACS in resting EEG while theta power in frontal areas was enhanced.

Based on the findings reviewed above, the present study examines whether the boosting of theta activity by 6 Hz electrical stimulation over the rIFG reduces the number of errors as well as the reaction time in a response inhibition task during and after stimulation. For this purpose, 6 Hz tACS was applied over right IFG during a motor response inhibition task (Go/NoGo). To examine whether the effects of the 6 Hz tACS was specific for the applied frequency, a full spectrum random noise stimulation (tRNS) was added as a second stimulation condition. In contrast to tACS, tRNS applies alternating electrical currents of different frequencies and amplitudes (for an overview, see [18, 24] and might hence be implemented as a control condition.

We hypothesized that the tACS at theta (6 Hz) during the task might reduce errors and reaction times in comparison to sham and tRNS stimulation.

## 2. Materials and Methods

The study was carried out in accordance with the latest revision of the Declaration of Helsinki. Experimental procedures were approved by the local ethics committee of the Christian Albrechts University, Kiel, Germany. Prior to the experiment, subjects gave their written informed consent.

### 2.1. Subjects

With G^∗^Power [25], a sample size of 20 subjects was calculated for a 2 × 3 way ANOVA with repeated measures. Used parameters were an effect size of *f* = 0.3 (estimated based on Polania and colleagues [16], Iuculano and Cohen Kadosh [26], and van Driel and colleagues [27]), correlation of the repeated measures of *r* = 0.5, *α* = 0.05, and a power of 0.80. Twenty-three healthy right-handed adults (age mean 22.91 years, range 18–30; 16 females) with normal or corrected to normal vision participated after giving informed consent. All participants were university students and were recruited via social media and flyers at the Kiel University. Exclusion criteria were (1) history or family history of epileptic seizures, (2) history of migraine, (3) unexplained loss of consciousness or brain related injury, (4) history of other neurological or psychiatric disorders, (5) cardiac pacemaker or intracranial metal implantation, and (6) intake of central nervous system-effective medication.

To exclude persons with psychological problems, the self-report questionnaire Symptom-Checklist-90-R/SCL-90-R [28] was used. Additionally the degree of ADHD symptoms was assessed with the screening questionnaire ADHS-E [29] and the severity of a depression with the revised version of the Beck Depression Inventory (BDI) II [30]. To assess the handedness of the subjects, the Edinburgh Inventory was used [31]. All these questionnaires were completed according to manual and at the end of the first session (see Table 1 for subject characteristics). Additionally, for each of the sessions, smoking and caffeine were assessed and protocolled.

After finishing each experimental session, the participants were asked to complete a questionnaire on the side effects of the stimulation, adapted from Poreisz and colleagues [32].

The questionnaire contains items pertaining to the presence and severity of headaches, change or difficulties in concentration, mood, visual perception, presence of fatigue, and discomforting sensations like pain, tingling, itching, or burning. The participants received one cinema voucher per session for their participation.

### 2.2. The Go/NoGo Task

Subjects performed a Go/NoGo task and responded by pressing a mouse button with their right forefinger. During the paradigm, single white digits between 1 and 9 were presented on a black background. The task was presented in two blocks of 600 trials each, of which 120 (20%) were NoGo trials. Subjects were asked to respond to stimuli as quickly as possible by pressing a button as soon as a digit between 1 to 4 and 6 to 9 appeared (“Go” stimuli) and were told to withhold a button press when a “5” appeared (“NoGo” stimuli). Participants were instructed to keep their eyes focused on the fixation cross and to avoid any movement during the acquisition. Each stimulus was displayed for 0.2 s with an average interstimulus interval of 1.45 s (randomly jittered between 1.3 and 1.6 s). The fixation cross at the center of the screen was constantly visible during the interstimulus interval. The term “hit” will subsequently be used to refer to button presses during a Go trial, while the term “false alarm” will refer to commission errors, i.e., button presses during a NoGo trial. The term “correct withhold” will be used to describe correctly withholding button press in NoGo trials, and “miss” describes when the button is not pressed during a Go trial. The subjects were seated 63 cm away from the monitor (37.6 cm × 30 cm; 19 Zoll); stimuli were controlled via the presentation software version 19.0, Neurobehavioral Systems Inc., Berkeley, CA, http://www.neurobs.com).

### 2.3. Brain Stimulation

Stimulation was performed with the NeuroConn DC-Stimulator (NeuroConn GmbH, Ilmenau, Germany) using 5 × 5cm sponge electrodes. The active electrode was placed at the crossing point of T4-Fz and F8-Cz according to EEG 10–20 System [12]. The return electrode was placed over the left supraorbital cortex. Theta tACS was performed at a frequency of 6 Hz; in the tRNS condition, full spectrum tRNS (0.1–640 Hz) was used. Current intensity was 1 mA and stimulation lasted 20 min. Ramping at the beginning and the end of the stimulation was 10 s in all conditions. In the sham condition, 30 s of 6 Hz tACs was applied at the beginning.

### 2.4. Experimental Design

In these two factorial repeated measures designs, subjects took part in all the conditions, each one week apart. Conditions were randomized, and subjects were blinded to the stimulation condition.

**Figure 1:**
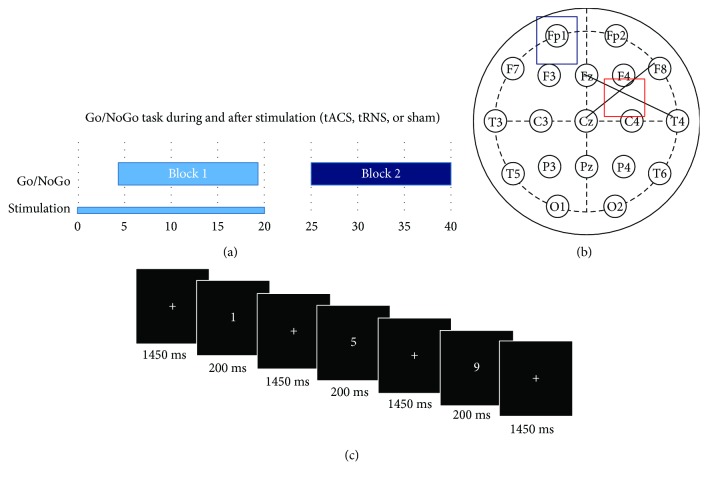
Experimental design. (a) Time course of the experiment: in each of the sessions, the subjects completed a 2 min practice and afterwards two blocks of 15 min each of a Go/NoGo paradigm. Stimulation took place during the first block of the paradigm, followed by a resting period of 5 min and the second block without stimulation. The task started 4.5 min after the beginning of the stimulation. (b) Electrode setup: active stimulation (5 × 5 cm) electrode was placed above rIFG, which was identified as the crossing point between T4-Fz and F8-Cz. The return electrode (5 × 7 cm) was placed above Fp1 following the international 10–20 system. (c) Go/NoGo task: subjects were asked to respond to stimuli as quickly as possible by pressing a button as soon as a digit between 1 to 4 or 6 to 9 appeared (“Go” stimuli) and were told to withhold a button press when a “5” appeared (“NoGo” stimuli). Participants were instructed to keep their eyes focused on the fixation cross and to avoid any movement during the acquisition. Each stimulus was displayed for 0.2 s with an average interstimulus interval of 1.45 s (randomly jittered between 1.3 and 1.6 s).

In order to keep the performance of the subjects as comparable as possible, the experimental sessions were performed at the same time of the day. In each of the sessions, the subjects completed a 2 min practice and afterwards two blocks of 15 min each of a Go/NoGo paradigm. Stimulation took place during the first block of the paradigm, followed by a resting period of 5 min and the second block without stimulation. So, the task was first performed online stimulation and afterwards offline. To avoid an influence of negative sensations during the ramping of the stimulation on the task performance, the task started 4.5 min after the beginning of the stimulation (see Figure 1).

## 3. Data Analysis and Statistics

### 3.1. Behavioral Data

Statistical analysis was done with R [33] and visualization with prism (GraphPad Prism version 5.00 for Windows, GraphPad Software, San Diego, California, USA).

Errors were analyzed by calculating the relative frequency of reactions on NoGo stimuli (false alarms). Number of errors and the relative frequency of errors were not normally distributed and therefore logarithmized for parametric analyses [34]. To avoid nondefined values, relative frequencies of a value of 0 were replaced by 0.001 and of the value 1 by 0.999 [35]. Accuracy was calculated as the quotient of all correct responses (hits and rejections) and to every response. Reaction times were analyzed based on the median. For the relation of reaction times and numbers of errors, the “Inverse Efficiency Score” (IES) was calculated [36]. The IES is the quotient of the median of the reaction times of hits of a subject and the accuracy. This score is especially useful for tasks with very low error rates of up to 10% [37], which applies for the Go/NoGo task. To analyze the effects of stimulation and the time point of it on reaction times, errors and the IES 2 × 3 repeated measures ANOVAs with the within factor stimulation (6 Hz tACS vs. tRNS vs. sham) and time of testing (during stimulation vs. after stimulation) were calculated. Normal distribution was inspected with histograms. Sphericity was tested with the Mauchly's sphericity test. If sphericity was not fulfilled for a Greenhouse-Geisser epsilon < 0.75, *p* was corrected; according to Greenhouse-Geisser (*p*_(GG)_), if Greenhouse-Geisser epsilon was >0.75, *p* was corrected according to Huynh-Feldt (*p*_(HF)_). This correction was necessary in all three ANOVAs. In case of significant interaction effects (stimulation × time) or main effects of stimulation, additional exploratory *t* tests were performed and *p* values were compared to Bonferroni-adjusted alpha levels *α*/3 = 0.0167 for three comparisons (global alpha level for the *t* tests was *α* = 0.05). Additionally, Bayes factors (BFs) were calculated to obtain more precise evidence on the hypothesis H0 [38, 39].

### 3.2. Adverse Event Questionnaire

For each side effect, the occurrence (yes/no) and severity (Likert scale: 1 mild–5 extremely high intensity) were checked. The number of adverse effects as well as the severity of each adverse effect were not normally distributed (Kolmogoroff-Smirnoff test) and therefore compared using the Mann–Whitney *U* tests. Statistical significance was defined as a two-tailed *p* value of less than 0.05.

## 4. Results

None of the subjects requested to terminate stimulation or asked for any medical intervention during or after the end of stimulation.

### 4.1. Behavioral Data

**Figure 2:**
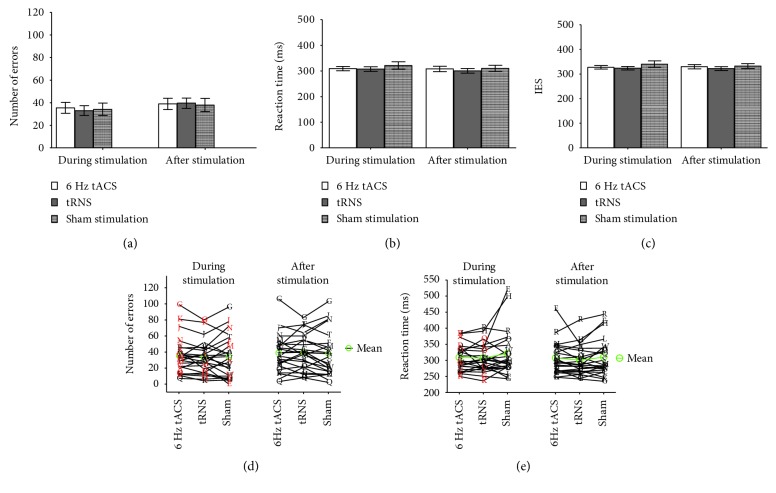
Behavioral data. In an 2 × 3 ANOVA with repeated measures, there was no significant interaction effect between stimulation and time of testing for number of false alarm trials (a) as well as reaction time for hit (b). (c) Inverse Efficiency Score. Also for the IES, there were no significant interaction effects between stimulation condition and time point. In (a), (b), and (c), means and standard deviations are reported. (d) and (e) Variability of stimulation. Each letter corresponds to one subject. The red color signifies that subjects identified the stimulation correctly.

In a 2 × 3 ANOVA with repeated measures, there was no significant interaction effect between stimulation and time of testing on the numbers of committed inhibition errors (*F*_(2,44)_ = 1.05, *p*_(HF)_ = 0.345, *η*^2^ = 0.05, and BF_01_ = 7.90). There was no main effect of stimulation (*p* = 0.136 and BF_01_ = 5.12), but an effect of time of testing (*p* = 0.006 and BF_01_ = 0.44) (Figure 2(a)). There is no significant interaction effect of stimulation and time of testing (*F*_(2,44)_ = 0.70, *p*_(HF)_ = 0.472, *η*^2^ = 0.03, and BF_01_ = 18.75) on reaction time (Figure 2(b)). Also for the IES, there were no significant interaction effects between stimulation condition and time of testing (*F*_(2,44)_ = 0.84, *p*_(GG)_ = 0.408, *η*^2^ = 0.04, and BF_01_ = 13.38). There was weak evidence for H0 for the effect of stimulation on IES (BF_01_ = 2.64). Evidence for H0 for the effect of stimulation on errors (BF_01_ = 5.12) and reaction time (BF_01_ = 6.9) as well as for time of testing on reaction time (BF_01_ = 3.48) and IES (BF_01_ = 17.93) was positive to strong (Figure 2(c)).

### 4.2. Adverse Event Questionnaire

**Figure 3:**
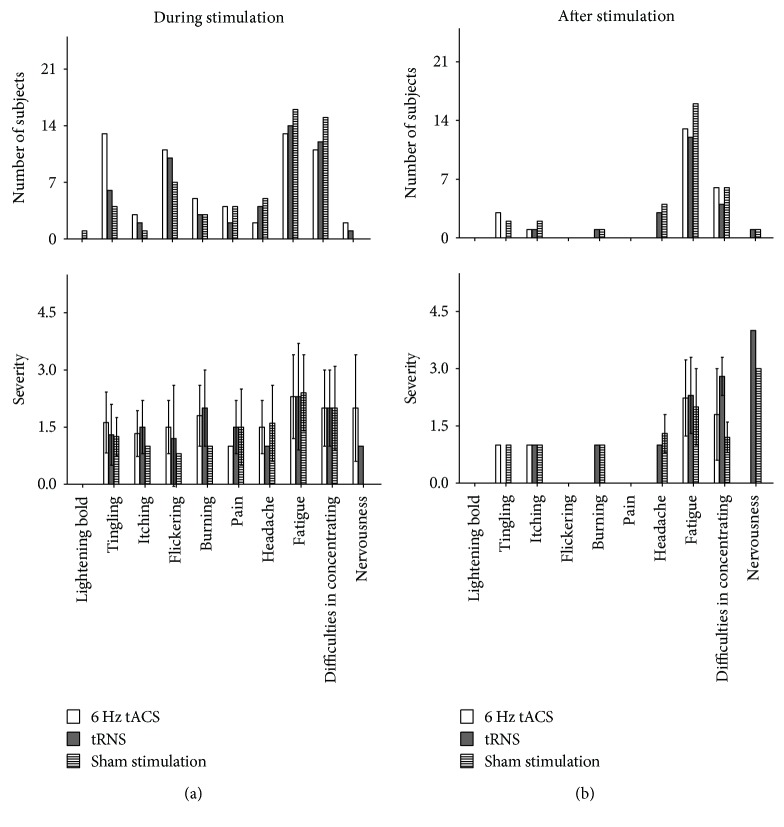
Adverse events during and after stimulation. (a) Mann–Whitney *U* tests showed a significantly higher incidence of tingling during 6 Hz tACS compared to tRNS (*p* = 0.039 and *U* = 184.0) and sham stimulation (*p* = 0.007 and *U* = 161.0). (b) There was a significantly higher incidence of tingling during compared to after stimulation for 6 Hz tACS (*p* = 0.0023 and *U* = 149.5).

Figure 3 summarizes the adverse events during and after stimulation. Generally, it can be said that Mann–Whitney *U* tests showed a significantly higher incidence of tingling during 6 Hz tACS compared to tRNS (*p* = 0.039 and *U* = 184.0) and sham stimulation (*p* = 0.007 and *U* = 161.0). However, concerning the intensities (NAS 1–5) of the observed side effects, there was no significant difference between the stimulation conditions.

None of the subjects could distinguish between active and sham stimulations. The order of sessions had no effect on guess rate concerning the experimental condition. Sham stimulation as well as 6 Hz tACS and full spectrum tRNS were indistinguishable regarding side effects. Thus, the blinding procedure was judged as being successful.

### 4.3. Comparisons between the Side Effects during and after Stimulation

Mann–Whitney *U* tests showed a significantly higher incidence of tingling (*p* = 0.0023 and *U* = 149.5) during stimulation compared to the data obtained after for 6 Hz tACS. However, concerning the intensities (NAS 1–5) of the observed side effects, there was no significant difference between the two time points.

## 5. Discussion

In the present study, we investigated the effect of transcranial alternating current stimulation on response inhibition in healthy young adults hypothesizing that tACS at theta (6 Hz) during the task will improve performance (errors and reaction times) in comparison to sham and transcranial random noise stimulation. As some studies report an improvement of performance not during stimulation but afterwards [40], we measured participants' Go/NoGo performance both while receiving stimulation (“online”) and following the stimulation (“offline”).

Based on the behavioral data in this study, we could not show effects of 6 Hz tACS and tRNS on response inhibition in healthy adults: both kinds of stimulation did not result in an improvement of performance such as less errors or faster reaction times compared to the tRNS and sham condition either during stimulation or after it.

Previous studies support the relevance of the rIFG for response inhibition [2, 41, 42]. However, by stimulating that structure with 6 Hz tACS, we were not able to modulate response inhibition. Therefore, our findings indicate the importance of region and frequency specific stimulation. It would be interesting to identify external and internal factors that might account for the negative results. Below, we discuss possible underlying mechanisms behind our negative findings in light of previous studies in the field.

### 5.1. Stimulation Parameters and Differences to Previous tES Studies

As mentioned above, anodal tDCS to the right inferior frontal gyrus (rIFG) improves behavioral inhibition [12, 13]. Compared to tDCS, a different mechanism is at work during tACS, requiring a different rationale for designing an experiment. It is especially crucial to identify a cognitive process that is characterized by a specific brain oscillation or combination of oscillations [18].

Even though behavioral outcomes of tACS have been demonstrated successfully, the underlying mechanisms have not been fully explored. While it is often hypothesized that tACS can phase-align neural oscillations, it is still relatively unclear whether it is able to modulate oscillatory power (for review, see [40, 43]). Only few studies have proven to enhance the power of a certain frequency band [14, 44]. Therefore, the applied tACS in our study might have affected only the oscillatory phase but not power, thus leading to our lack of significant effects.

Theta activity frequency used in this study ranges from 4 to 8 Hz and the tACS with 6 Hz frequency might not be suited to modulate theta activity in rIFG. Therefore, the exact parameters of the stimulation play an important role and still need to be optimized. However, it was shown that 6 Hz tACS over tempo parietal cortex can enhance cognition in older adults [21].

We applied continuous theta tACS, even though the target theta activity occurred only after an error. During a Go/NoGo task, different attentional processes are relevant, including not only response inhibition but also other processes such as error monitoring. The task also requires sustained attention, which has been proven to be accompanied by a decrease of theta activity in medial frontal cortex [45, 46]. So therefore, the ongoing theta tACS might have enhanced theta activity where it was counterproductive to the performance in the Go/NoGo task. Chander and colleagues [47] showed that theta band tACS during a working memory task disrupted the task performance; this was associated with decreased frontal midline theta amplitude.

tACS is assumed to entrain spontaneous oscillations but the effect of theta power during response inhibition involves induced oscillations (for review, see [18, 48]). If spontaneous and induced oscillations involve two different mechanisms, tACS might only be a promising technique to manipulate spontaneous oscillations.

Previous studies indicate that state-triggered and closed loop stimulation boosts effects of noninvasive transcranial brain stimulation (for review, see Karabanov et al. [49]); e.g., in patients with Parkinson's disease, tACS applied to the motor cortex in their individual tremor frequency was able to suppress tremor amplitude. To maintain the optimal phase delay between tACS and the endogenous tremor rhythm, the phase of tACS was constantly adjusted, informed by the ongoing tremor activity [50]. In contrast, in our study, the phase of the 6 Hz tACS signal was not adjusted to the phase of the endogenous brain oscillations and was therefore independent. Therefore, closed loop stimulation might be more effective for specific modulation of brain function.

Another interesting finding is the negative effect of tRNS; as mentioned above, we use full spectrum tRNS (from 0.1 to 640 Hz) as a second condition to examine whether the effects of the 6 Hz tACS are specific for this frequency. tRNS is a relatively new form of brain stimulation, and studies have already shown how it can promote and sustain perceptual learning [51–53] besides its clear modulatory effects on the motor cortex [54]. However, higher frequencies (100–640 Hz) and not frequencies less than 100 Hz were responsible for this excitability increase (for review, see Antal and Paulus [55]).

Furthermore, different methodological factors (i.e., stimulation montage, intensity, and frequency of current) affecting specific brain networks interact with ongoing neural processes and transcranial brain stimulation effects also depend on the specifics of the experimental design [10, 17, 56] (for review, see Antal and Herrmann [18]).

Another possible explanation for the negative finding in our study could be the size of the electrodes (5 × 5 cm); the behavioral effects of transcranial electrical stimulation appear to be also critically dependent on the position of the return electrode. Changing the locations of the electrodes has been shown to alter the electric field in the brain [57–63]. Effects of tDCS and tACS also depend on the orientation of both the stimulating and reference/return electrodes altering the electric field in the brain; electrode size determines the extent of the stimulated area under the electrodes [64]. Our study used smaller active electrodes with a noncircular surface compared to many previous studies that stimulated the same area of interest using tACS/tRNS with larger or circular stimulation electrodes [21, 65, 66].

### 5.2. Individual Differences

Our negative results could also reflect in part the substantial individual variation in response to tES. Previous studies point out that the effectiveness of stimulation depends on initial brain state, for which baseline performance is a crude yet valuable indicator [67, 68] Morphological and anatomical characteristics determine the field amplitude of applied current by using the same electrode montages as demonstrated by tDCS study of Parazzini and colleagues, who compared the effects of four different electrode montages on three different head models [69]. Besides individual's anatomical features, there are much more determinants, like age, sex circadian rhythm, and hormonal levels to take into account [68, 70–72].

So far, most studies on physiological effects of tACS rely on aftereffects of the stimulation and resting-state measurements while research on behavioral effects of tACS mainly focused on online effects of the stimulation [14, 44, 73, 74].

Despite there being no behavioral online or offline effects of 6 Hz tACS as well as tRNS in our study, on a descriptive level, there was a tendency towards a change in the response behavior both in the control conditions (higher number of errors in the tRNS and shorter reaction times in the sham and tRNS condition) (Figures 2(d) and 2(e)). Yet, this effect did not yield statistical significance. Analysis of the behavioral response showed that 18 of the 23 subjects (72%) were either faster or produced less errors in the 6 Hz condition compared to sham, but did not improve on both. The reason of this result could lie in individual response strategies. Also, it is possible that the stimulation over rIFG reached different regions of rIFG in the different subjects. Hampshire and colleagues [75] suggest that two functionally distinct subregions of the rIFG might exist, one region being involved in attention while another region being involved in inhibition. Since the anatomy of rIFG is very complex, the stimulation could have led to an improvement of reaction times or committing less inhibition errors, depending on the region reached by the stimulation. This is supported by the findings in previous tDCS research, where different montages have resulted in an improvement of either reaction times or errors [12, 13, 76–78].

It is possible that tACS will not increase theta activity upon a certain level and therefore will not lead to behavioral benefits in people who do not lack theta activity, whereas it might induce improvement in people with a deficit of theta activity. A comparable effect was shown by Herrman and colleagues [43] for alpha activity: alpha activity was only increased by stimulation with the individual alpha frequency (IAF) of the subject, when the IAF power had been low beforehand. If the IAF power had been high before stimulation, no additional increase was found. So even though our hypothesis in this study could not be proved in healthy young adults, it should be further investigated in persons with ADHD as the power of their individual theta frequency is expected to be lower [7]. Also, it is possible that, while being absent on a behavioral level, changes were induced on a neurophysiological level.

Additionally, the brain-derived neurotrophic factor (BDNF) polymorphism is suggested to have an impact on transcranial stimulation-induced plasticity in humans, which differs according to the mechanism of plasticity induction [79].

### 5.3. Suitability of the Paradigm

Van Boxtel and colleagues have discussed whether “there is a centrally located inhibitory mechanism” [80], which “suppresses irrelevant responses” and whether “inhibition is localized to right IFG alone” [3]. rIFG is known to be important in different inhibition paradigms besides the Go/NoGo, e.g., the “stop after go” paradigm. Depending on the paradigm, different networks are activated during motor inhibition. Comparing the activation in a stop signal, Go/NoGo, and antisaccade paradigm, pre-SMA and different regions of IFC are activated, but still they are related to an activation of rIFG [2]. We decided to use the Go/NoGo paradigm to be able to compare our results to those of previous studies [6].

Early findings by Menon and colleagues [81] have provided evidence for a distributed error processing system in the human brain that overlaps partially with brain regions involved in response inhibition and competition. The authors found that the IFC is activated both during response inhibition and error processing. Since in the study of Mazaheri et al. [6], response inhibition and error monitoring are combined, possibly stimulating the IFC, rather than the rIFG would lead to an improvement in the Go/NoGo task.

Task difficulty is also another contributor to the state-dependent nature of the effects of tES [82]. For healthy students, our Go/NoGo task is a relatively easy and simple task. Therefore, ceiling effects may have masked any change in performance. Yet, subjects made relatively high number of errors, possibly due to the comparatively short and jittered response window and the long duration of the task, leaving sufficient room for improvement in most subjects.

## 6. Conclusion Suggestions for Improvements

Based on behavioral data, we could not show effects of 6 Hz tACS and tRNS on response inhibition in healthy adults. The lack of the well-described effect supports the notion that the setup of our montage and paradigm might not have been effective in showing improved performance during and after 6 Hz tACS.

A behavioral baseline would be an additional possibility to control for intraindividual differences. Yet, the lack of a baseline measure in our study poses a clear limitation. However, our within-subject design enables the comparison to a sham condition as a control condition with sessions at the same time of the day.

To determine whether the negative results translate to a larger population, a higher number of participants is required. The results derived from our study could contribute to the exploration of effects of noninvasive transcranial brain stimulation in a healthy population and should help to optimize existing stimulation protocols. Further studies should include simultaneous EEG recordings to investigate the underlying neurophysiological processes. Sharing negative findings will have (1) positive impact on future research questions and study design and (2) will improve knowledge acquisition of noninvasive transcranial brain stimulation techniques.

## Figures and Tables

**Table 1:** Subject characteristics.

	Mean ± standard deviation	Exclusion criteria
Sex	16 females, 7 males	
Age	22.91 years ± 3.44	18 < age > 30
Edinburgh Handedness Inventory laterality quotient (HQ)	80.99 ± 22.46	HQ < 50
BDI II total score	4.52 ± 3.36	BDI > 13
ADHS-E percentile rank	58.48 ± 23.13	PR > 98
SCL-90-R *T* value GSI	45.78 ± 7.89	*T* > 65
SCL-90-R *T* value PST	47.17 ± 6.86	*T* > 65
SCL-90-R *T* value PSDI	46.70 ± 11.65	*T* > 65

Data are presented in M ± SD. No subject had to be excluded because of these exclusion criteria.

## Data Availability

The behavioral data used to support the findings of this study are available from the corresponding author upon request.
